# Clinical and dosimetric dataset of time-to-event normal tissue complication probability for osteoradionecrosis

**DOI:** 10.1038/s41597-025-06321-w

**Published:** 2026-01-09

**Authors:** Natalie A. West, Serageldin Kamel, Andrew Wentzel, Zaphanlene Kaffey, Moamen Abdelaal, G. Elisabeta Marai, Guadalupe Canahuate, Xinhua Zhang, Melissa M. Chen, Kareem A. Wahid, Jillian Rigert, Kristy K. Brock, Mark Chambers, Adegbenga O. Otun, Ruth Aponte-Wesson, Renjie He, Mohamed A. Naser, Katherine A. Hutcheson, Abdallah S. R. Mohamed, Lisanne V. van Dijk, Amy C. Moreno, Stephen Y. Lai, Clifton D. Fuller, Laia Humbert-Vidan

**Affiliations:** 1 The University of Texas MD Anderson Cancer Center, Houston, TX USA; 2 The University of Texas MD Anderson UTHealth Houston Graduate School of Biomedical Sciences, Houston, TX USA; 3 University of Illinois Chicago, Chicago, IL USA; 4 Emory University, Atlanta, GA USA; 5 University of Iowa, Iowa City, IA USA; 6 Baylor College of Medicine, Houston, TX USA; 7 University Medical Center Groningen, Groningen, Netherlands; 8 Vall d’Hebron Institute of Oncology, Barcelona, Spain

**Keywords:** Head and neck cancer, Cancer models, Outcomes research, Risk factors, Oral manifestations

## Abstract

Osteoradionecrosis of the jaw (ORNJ) is a radiation-induced late toxicity that can dramatically decrease patients’ quality of life. Recent increases in survival rates of head and neck cancers associated with human papillomavirus (HPV) infection have resulted in a higher frequency of radiation-induced toxicities, particularly ORNJ. Recent work with Normal Tissue Complication Probability (NTCP) models and a Weibull Accelerated Failure Time (WAFT) model have further developed our understanding of ORNJ clinical/dosimetric risk factors and longitudinal features, respectively. In this data descriptor, 1129 head and neck cancer (HNC) patients received curative intent radiotherapy (RT) at MD Anderson Cancer Center and were followed up with clinical and radiological assessments at 3–6, 12, 18, 24 months, and then annually following the conclusion of RT for development of ORNJ. This data, in addition to the patients’ demographic, supplementary clinical, and dosimetric information was recorded in a comma-separated value file embedded within this data descriptor. This large, longitudinal dataset is a significant resource for further systematic analysis of post-RT normal tissue outcomes in HNC.

## Background & Summary

Head and neck cancers (HNC) affect over 58,000 Americans annually, with a growing proportion attributed to human papillomavirus (HPV) infection^1^. HPV-associated HNC are notably diagnosed in younger populations and are associated with higher survival rates in comparison to HPV-negative HNC^2^. Radiation therapy (RT) remains the mainstay of treatment for HPV-positive HNC, but the combination of RT with extended survival has led to an increased incidence of RT-induced late toxicities in normal tissues. One such complication is osteoradionecrosis of the jaw (ORNJ), a severe sequela following RT with an incidence ranging from 4 to 15%^3^. The mechanism of ORNJ is believed to be first instigated by compromised vascularity through hypoxic, hypovascular, and hypocellular tissue (Marx’s 3 H’s)^4^ followed by progressive loss in cortical bone integrity, ultimately impairing oral function and quality of life^5,6^. Due to the favorable RT response and prognosis of HPV-associated HNC and the subsequent number of patients transitioning to survivorship, there is a need to better understand the timing and progressive risk of ORNJ in relation to radiation treatment of HNC in order to optimize prevention efforts of this often debilitating condition.

Previous cross sectional statistical analyses^7–10^, including Normal Tissue Complication Probability (NTCP) models of ORNJ^11,12^, have identified clinical and dosimetric risk factors associated with this sequela. Additionally, some studies have explored statistical correlations on longitudinal ORNJ data^13,14^. In recent work, we developed a fully parametric multivariable Weibull Accelerated Failure Time (WAFT) model to predict patient-specific ORNJ risk over time based on longitudinal data.

This data descriptor presents the underlying dataset used for the development of the ORNJ WAFT model^15^. The dataset is comprised of a large, longitudinal cohort of HNC patients and includes detailed demographic, clinical, and dosimetric variables, along with structured follow-up data and time-to-ORNJ events. The availability of this dataset offers a valuable resource for modeling ORNJ and supports the development of predictive tools for personalized survivorship care in HNC.

## Methods

### IRB protocol

After the University of Texas MD Anderson Cancer Center Institutional Review Board approval, data were extracted from a philanthropically funded observational cohort at the University of Texas MD Anderson Cancer Center (Stiefel Oropharynx Cancer Cohort, PA14-0947). A waiver of informed consent was approved through the MD Anderson RCR030800 protocol, allowing for retrospective analysis. All patients included were consented RT cases. We implemented formal reporting guidance as per Enhancing the QUAlity and Transparancy Of health Research Network guidance, using *the RECORD Statement*^16^, attached as a supplement.

### Patient population

1129 HNC patients from an internal MD Anderson Cancer Center cohort were treated with curative intent RT from 2005 to 2022. Patients were closely followed via clinical and radiological assessments every 3, 6, 12, 18, and 24 months, and then approximately annually following the conclusion of RT. As this cohort derives from a single institution, generalizability to institutions with different patient demographics and treatment practices may be limited. This dataset has been externally validated on an independent cohort in the parent study^15^. The patient data was stored in and accessed via the Epic Electronic Health Record System.

### Demographic data

All demographic, clinical, and dosimetric variables are summarized in Table 1. The patients’ demographic data included: gender (male or female), age (in years), smoking status (current, former, never), and smoking pack-years. Smoking pack-years were calculated by the product of tobacco packs smoked per day and number of years smoked (Table 2).

**Table 1:** Demographic, clinical, and dosimetric data for included patient cohort showing variable, its respective coding (qualitative or quantitative) and units (when applicable).

Data Classification	Variable	Code	Units
Demographic Data	Gender	Male	N/A
		Female	
	Age	Quantitative	Years
	Smoking Status	Current smoker	N/A
		Former smoker	
		Never smoker	
	Smoking Pack-Years	Quantitative	Pack-years
Clinical Data	Overall Survival	0 - Deceased at last F/U	N/A
		1 - Alive at last F/U	
	ORNJ Status	0 - Negative for ORNJ	N/A
		1 - Positive for ORNJ	
	Time to Event	Quantitative	Months
	ORNJ Grade*	1 - Minimal bone exposure, conservative management only	N/A
		2 - Minor debridement received	
		3 - HBO needed	
		4 - Major surgery required	
	Pre-RT Dental Extractions	0 - No pre-RT dental extractions	N/A
		1 - Pre-RT dental extractions	
	Chemotherapy	Induction	N/A
		Induction and concurrent	
		Concurrent	
		No chemotherapy	
	Postoperative RT vs. Definitive RT	Postoperative RT	N/A
		Definitive	
	HPV/p16 + Ve Status	Yes	N/A
		No	
		Unknown	
	Tumor Site Group	Oropharynx	N/A
		Oral cavity	
		Nasopharynx/nasal cavity/paranasal sinuses	
		Larynx/hypopharynx	
		Other	
	Mandible Volume	Quantitative	cm^3^
Dosimetric Data	V5-80 Gy	Quantitative	cm^3^
	D0.5%-99.5%	Quantitative	Gy

This can be used to supplement the CSV file included within this data descriptor.

*Adapted from Tsai staging system.

F/U = follow-up. HBO = hyperbaric oxygen. Tumor Site Group – Other = pharynx and oral cavity other.

**Table 2:** Distribution of demographic and clinical data stratified by ORNJ status (control group and ORNJ group).

Variable		Amount of Control (n = 931)	Amount of ORNJ (n = 198)
Gender	Male	764 (82.1%)	174 (87.9%)
	Female	167 (17.9%)	24 (12.1%)
Median Age (IQR)		60.0 (14.0)	60.0 (12.0)
Primary Tumor Site	Oropharynx	602 (64.7%)	138 (69.7%)
	Oral Cavity	156 (16.8%)	49 (24.8%)
	Larynx/Hypopharynx	187 (20.1%)	7 (3.5%)
	Nasopharynx, Nasal Cavity, Paranasal Sinuses	21 (2.3%)	2 (1.0%)
	Other	5 (0.5%)	2 (1.0%)
T Stage	T0	33 (3.5%)	3 (1.5%)
	T1	210 (22.6%)	31 (15.7%)
	T2	314 (33.7%)	63 (31.8%)
	T3	200 (21.5%)	39 (19.7%)
	T4	160 (17.2%)	59 (29.8%)
	T4a	8 (0.9%)	3 (1.5%)
	T4b	0 (0.0%)	0 (0.0%)
	TX	6 (0.6%)	0 (0.0%)
N Stage	N0	189 (20.3%)	32 (16.2%)
	N1	210 (22.6%)	30 (15.2%)
	N2	205 (22.0%)	69 (34.9%)
	N2a	23 (2.5%)	2 (1.0%)
	N2b	181 (19.4%)	40 (20.2%)
	N2c	99 (10.6%)	21 (10.6%)
	N3	21 (2.3%)	4 (2.0%)
	NX	3 (0.3%)	0 (0.0%)
HPV+	Yes	174 (18.7%)	20 (10.1%)
	No	17 (1.8%)	2 (1.0%)
	Unknown	740 (79.5%)	176 (88.9%)
Smoking Status	Current	126 (13.5%)	33 (16.7%)
	Former	445 (47.8%)	93 (47.0%)
	Never	360 (38.7%)	72 (36.4%)
Pre-RT Dental Extraction*		231 (24.8%)	76 (38.4%)
Pre-RT Surgery	Postop RT	154 (16.5%)	43 (21.7%)
	Definitive RT	777 (83.5%)	155 (78.3%)
RT Technique	IMRT	678 (72.8%)	161 (81.3%)
	VMAT	188 (20.2%)	24 (12.1%)
	IMPT	17 (1.8%)	4 (2.0%)
	Non-IMRT	11 (1.2%)	0 (0.0%)
	N/A	37 (4.0%)	9 (4.6%)
Chemotherapy	Concurrent chemotherapy	465 (50.0%)	97 (49.0%)
	Induction and concurrent	202 (21.7%)	59 (29.8%)
	No chemotherapy	189 (20.3%)	24 (12.1%)
	Induction chemotherapy	75 (8.1%)	18 (9.1%)
Median follow up time in years (range)		8.0 (6.6)	8.0 (5.4)

Columns three and four demonstrate n number of patients represented per variable in each population (control or ORNJ) and their relative percent distribution.

*Only dental extractions within six weeks prior to the start date of the RT course were considered.

### Clinical data

The patients’ clinical data included: overall survival, ORNJ status (binary, yes or 1 vs. no or 0), time to event, ORNJ grade, pre-RT dental extractions, T stage, N stage, chemotherapy (induction vs. induction and concurrent vs. concurrent vs. no chemotherapy), post-operative RT vs. definitive RT, HPV/p16 + Ve status (yes vs. no or unknown), tumor site group (oropharynx vs. oral cavity vs. nasopharynx/nasal cavity/paranasal sinuses vs. larynx/hypopharynx vs. major salivary glands vs. other), and mandible volume (in cubic centimeters, cc). 916 patients (81%) were coded with an HPV/p16 + Ve Status of ‘Unknown.’ While this is reflective of practical limitations^17^, it may bias future analyses, as HPV/p16 status has been shown to impact survival rates and quality of life^2,18^. Multiple imputation serves as a potential strategy to derive missing HPV/p16 statuses^19,20^. Patients with missing data were not included in the original analysis^15^. Overall survival time, binarily coded as 0 for no survival and 1 for survival, represents the time in months between time of RT start date and time of death or time to last follow-up. As this dataset covers a wide range of years preceding ORNJ grading consensus^21^, ORNJ status was binarily coded to account for any variability across staging systems^22^. 0 indicated no ORNJ detected and 1 indicated an active ORNJ diagnosis (of any grade) at time of last follow-up. To reflect current clinical standards, ORNJ grade was also specified using a numeric value of 0–4 following the Tsai staging system^23^. For patients with active ORNJ, time to event is calculated in months from the RT start date to time of ORNJ diagnosis. For patients without an active ORNJ diagnosis, the time to event was censored to be the time in months from RT start date to either time of death or last follow-up. Pre-RT dental extractions were binarily coded—0 indicated negative and 1 indicated positive for pre-RT dental extractions. T stage and N stage indicate the cancer stage, following the standard TNM staging system by the American Joint Committee on Cancer (AJCC, 7^th^/8^th^ edition) and the International Union Against Cancer. Chemotherapy and post-operative RT vs. definitive RT indicate if RT was combined with another treatment; ‘concurrent chemotherapy’ indicates chemotherapy occurred simultaneously with RT while ‘induction chemotherapy’ indicates chemotherapy was completed before RT. Likewise, ‘post-op RT’ indicates RT was completed following surgery while ‘definitive’ indicates RT was completed without surgery. HPV/p16 + Ve indicates positive expression of HPV/p16 via ‘yes’, ‘no’, or ‘unknown.’ Mandible volume was reported (in cc) from delineated mandible contours; mandible bone was auto-segmented with a previously validated multiatlas-based auto-segmentation using commercial software ADMIRE (research version 1.1; Elekta AB, Stockholm, Sweden).

### Dosimetric data

The patients’ dosimetric data included the following dose-volume metrics: volume of the mandible receiving at least a specified dose (V5-V80 Gy in 5 Gy increments), and dose received by a specified volume of mandible (D0.5%, D1%, D2%, D3%, D5-D95% in 5% increments, D97%, D98%, D99%, D99.5%). These metrics were calculated directly from the radiation dose distribution DICOM files utilizing a Python-based software developed from core standards and software^24–30^, notably pydicom and RT Dose Module Attributes as specified in DICOM PS3.3, and tested in-house.

## Data Record

The complete comma-separated value (CSV) file containing demographic, clinical, and dosimetric data for the aforementioned patient population is publicly available on figshare^31^. This CSV file provides the unique opportunity for analysis of a large HNC cohort with detailed treatment-related information related to prevalence and timing of ORNJ.

**Fig. 1:**
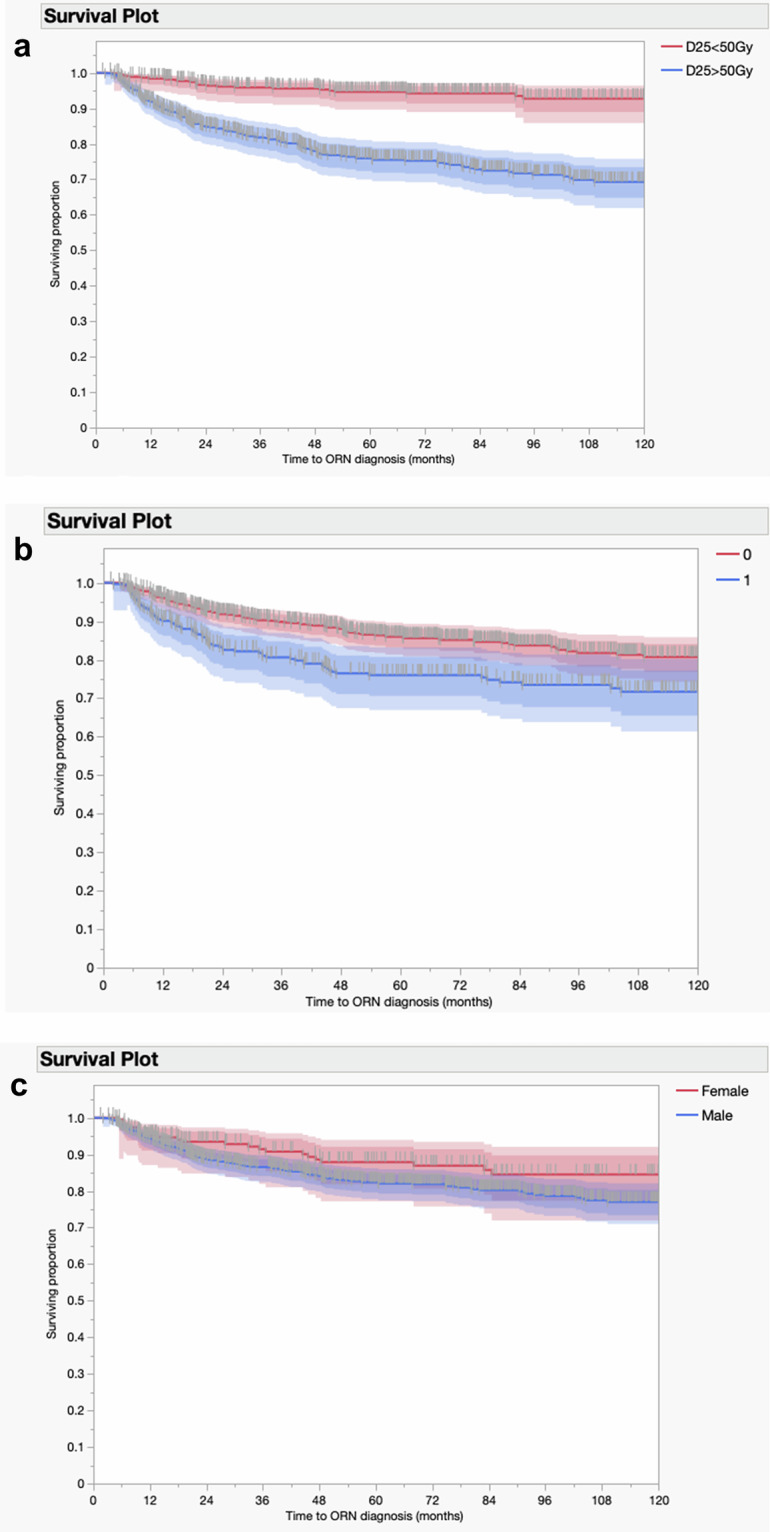
Right-censored Kaplan-Meier curves denoted time-to-ORNJ diagnosis, stratified by (**a**) D25% with a 50 Gy threshold, (**b**) dental extractions, with 0 and 1 indicating the absence or presence of a pre-RT dental extraction, respectively, and (**c**) gender.

**Fig. 2:**
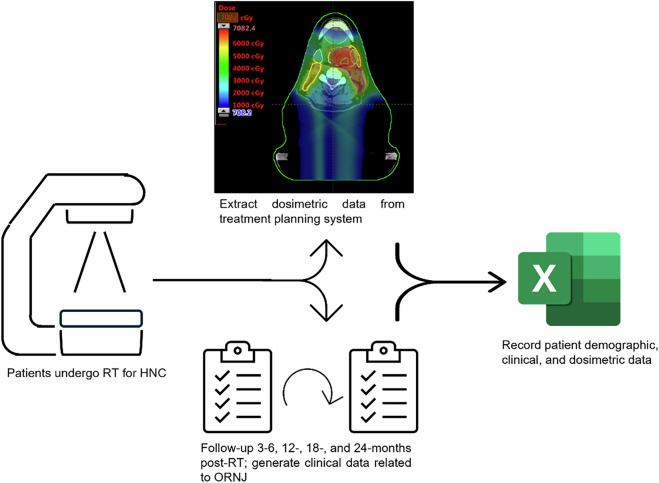
Diagram showing the data collection workflow and input into the final CSV file. Patients underwent RT at MD Anderson Cancer Center (left), in which dosimetric data was generated and acquired from a treatment planning system (top middle). Patient demographic and clinical data were also acquired from initial and follow-up visits (bottom middle). These data were then inputted into the CSV file included within this data descriptor (right).

The authors acknowledge the dichotomy of open science while maintaining patient confidentiality; this is particularly important with cohorts of long-term survivors. As such, patient identification was anonymized through a randomly assigned subject ID independent from their medical record number (MRN). The dataset contains no other patient identifiers (Figs. 1, 2).

## Technical Validation

Patient demographic and clinical data was stored and accessed via manual extraction by post-doctoral fellows with radiation oncology training from the University of Texas MD Anderson Cancer Center’s Epic Electronic Health Record System server and imported into REDCap electronic data capture tools hosted at the University of Texas MD Anderson Cancer Center^32,33^. The dataset was curated by multiple observers over time using a standardized template and variable dictionary. When discrepancies were suspected, records were double-checked against the original sources and corrected if inconsistencies were identified. Although formal inter-rater reliability statistics were not calculated, this approach provided additional quality assurance during the curation process to minimize misclassification and confirmation biases^34^.

Dosimetric data was obtained from clinical radiotherapy treatment plans using the RayStation treatment planning system (RaySearch Laboratories AB, Stockholm, Sweden). These data were first exported in standardized DICOM-RT (Digital Imaging and Communications in Medicine – Radiation Therapy) format and then analyzed to calculate dose-volume metrics to be used in the model.

1471 patients were examined for eligibility for this analysis. 342 patients were excluded due to clinical reasons such as prior irradiations; others were excluded for incomplete or missing data. The final cohort included a dataset of 1129 HNC from MD Anderson Cancer Center.

## Usage Notes

The WAFT-based time-to-ORNJ online calculator graphical user interface (GUI) is available at https://uic-evl.github.io/OsteoradionecrosisVis/.

## Supplementary information

RECORD Checklist

## Data Availability

The dataset is available on figshare^31^, publicly accessed at 10.6084/m9.figshare.26240435.v1. In accordance with NOT-OD-21-013, final NIH Policy for Data Management and Sharing, anonymized/de-identified data that support the findings of this study are openly available in an NIH supported generalist scientific data repository (figshare) no later than the time of an associated publication.

## Code availability

The script used for analyzing this dataset and training and testing the WAFT model can be found here in this repository: https://github.com/LaiaHV-MDACC/ORN-time-to-event-prediction-modelling.
